# Analysis on the epidemiological and drug resistance characteristics of osteoarticular tuberculosis in South-central China

**DOI:** 10.3389/fpubh.2024.1432071

**Published:** 2024-08-30

**Authors:** Tanwei Fang, Shuliu Yang, Binbin Liu, Wenbin Li, Qing Sun, Haican Liu, Yanyan Yu, Yu Xiang, Machao Li, Yi Guo, Jixiang Li, Xiuqin Zhao, Li-li Zhao, Kanglin Wan, Guilian Li, Xiuqin Yuan, Yunhong Tan

**Affiliations:** ^1^Hunan Institute for Tuberculosis Control & Hunan Chest Hospital, Changsha, China; ^2^National Key Laboratory of Intelligent Tracking and Forecasting for Infectious Diseases, National Institute for Communicable Disease Control and Prevention, Chinese Center for Disease Control and Prevention, Beijing, China; ^3^School of Public Health, University of South China, Hengyang, China; ^4^Department of Medicine, Hunan Traditional Chinese Medical College, Zhuzhou, China; ^5^Institute of Reproduction and Stem Cell Engineering, School of Basic Medical Sciences, Central South University, Changsha, Hunan, China

**Keywords:** osteoarticular tuberculosis, epidemiology, clinical characteristics, drug resistance, multi-drug resistance

## Abstract

**Objective:**

Osteoarticular tuberculosis (OATB) is one of the most common forms of extrapulmonary tuberculosis; however, limited epidemiological data are available on this public health concern worldwide, especially in developing countries. This study aimed to analyze the clinical epidemiology and drug resistance characteristics of OATB cases in Hunan province which located in South-central China.

**Methods:**

We retrospectively enrolled OATB patients with *Mycobacterium tuberculosis* culture positive at Hunan Chest Hospital from January 2013 through March 31, 2022. The multiple demographic, clinical variables and drug susceptibility data of the patients were collected from the hospital’s electronic patient records. Descriptive statistical methods, Chi-square test and logistic regression analysis were employed as statistical methods.

**Results:**

Of the 269 OATB cases, 197 (73.23%) were males, 206 (76.85%) were farmers; patients’ ages ranged from 5 to 85 years, 57 (21.19%) aged at 20–29 years old and 52 (19.33%) aged at 60–69 years old. In terms of the disease, 177 (65.80%) had spinal TB with most occurrence in lumbar vertebrae (26.02%, 70/269), multiple spinal sites (18.96%, 51/269) and thoracic vertebrae (15.24%, 41/269). Outside of the spine, OATB mainly occurred in the lower limb (13.38%, 36/269). In terms of drug resistance, 40 (14.87%) and 72 (26.77%) were resistant to rifampicin (RFP) and isoniazid (INH) respectively; 38 (14.13%) were multi-drug resistant (MDR), and a total of 78 (29.00%) isolates were drug resistant. OATB patients aged 40–49 years old (compared to those aged ≥70 years) and from the west of Hunan province, China (compared to those from the center of Hunan) were at risk for developing RR/MDR (ORs were 5.057 and 4.942, respectively; 95% CIs were 1.009–25.342 and 1.458–16.750, respectively).

**Conclusion:**

In South-central China, OATB mainly affected males, farmers and those aged 20–29 and 60–69 years old. Spinal TB is prone to occur in the lumbar and multiple spinal sites. The resistance situation of OATB was serious, and people aged 40–49 years old and patients from the west of Hunan were risk factors of RR/MDR. All these findings will help to improve the prevention, diagnosis and treatment strategies of OATB.

## 1 Introduction

Tuberculosis (TB), a chronic infectious disease caused by the bacillus *Mycobacterium tuberculosis* complex (MTBC), is still one of the leading causes of death worldwide. According to the report of the World Health Organization (WHO), globally, an estimated 10.6 million people fell ill with TB and 1.3 million deaths due to TB in 2022, the number of new TB cases in China was as high as 748,000, ranking the third among the countries with high burden in the world (1). MTBC infection typically affects the lungs but can affect other sites of the body as well, so TB is always divided into pulmonary TB (PTB) and extrapulmonary TB (EPTB) according to the site of the lesion. Common EPTB includes osteoarticular TB (OATB), lymphatic TB, tuberculous meningitis, intestinal TB, skin tuberculosis, and so on (2–4).

OATB, with its incidence always following lymphatic TB among EPTB, mainly affects the hands, feet, spine, and large weight-bearing joints, such as the hips and knee, manifesting as joint pain, deformation and even disability, and having serious impact on the quality of patients’ life (5). OATB is not a notifiable infectious disease in China and in many other countries, its exact incidence is unknown. Several studies reported that OATB accounts for about 1–25% of TB and 3.5–41% of EPTB, with spinal TB being the most common, followed by other OATB (6–9). Drug resistance (DR) of OATB is also severe, some studies reported that the DR rates ranged from 27.68 to 29.05%, while the MDR rates from 4 to 12.5% and extensively DR rates from 1.2 to 2.21% (6, 10–12).

Hunan province, located in South-central China and as the seventh population province with a total population of 72.463 million (13), has the second most TB cases in China (14). In recent years, many measures have been used to prevent and control TB in the province, it was reported the TB incidence decreased by 18.0% from 81.0/100,000 in 2018 to 66.4/100,000 in 2022 (14). Some studies have reported the TB resistance characteristics in this area (11, 15, 16). However, to date, the prevalence and drug resistance characteristics of OATB in Hunan province remain unclear. Therefore, this study retrospectively collected demographic, clinical information and drug resistance profiles of inpatients diagnosed with OATB in the Hunan Chest Hospital, a provincial TB institute and clinical center in Hunan province, from 2013 to 2021 to explore its epidemiological and DR characteristics, which will help to comprehensively understand the characteristics of OATB and provide basic clues for the prevention and treatment of OATB.

## 2 Materials and methods

### 2.1 Ethics approval and informed consent

This study was performed according to the ethical guidelines of the Helsinki Declaration and was approved by the Ethics Committee of Hunan Chest Hospital (LS2021051807). The written informed consent from each of the patients involved was not required according to the Human Ethics Committee. All sensitive patient information was removed before analysis.

### 2.2 Source of patients and *Mycobacterium tuberculosis* isolates

The subjects of our study were inpatients diagnosed with OATB in the Hunan Chest Hospital from January 1, 2013 to March 31, 2022. Hunan Chest Hospital is a provincial institute and clinical center of TB, also delivers treatments for cardiac diseases and thoracic tumors, and has a total of 1,600 beds. We collected demographic and clinical information, including gender, age, occupation, place of residence, diagnosis and drug susceptibility data from the hospital’s electronic patient records. All sensitive information of the patient was removed before analysis.

The inclusion criteria: (1) Patients have tuberculous lesions in bones or joints, such as spine (including cervical vertebrae, thoracic vertebrae, lumbar vertebrae, sacral vertebrae), elbow joint, hip joint, ankle joint, knee joint, shoulder joint, rib, foot, breastbone, skull, sternoclavicular, etc., were all diagnosed as OATB, and all OATB patients with or without TB lesions in the other body sites were all included; (2) OATB patients with *M. tuberculosis* isolates. The exclusion criteria: (1) Patients who lived in other provinces; (2) Patients with incomplete data; (3) Patients with repeated hospital visit records in 12 months; (4) Patients without *M. tuberculosis* isolates.

For the aim of analyzing the drug susceptibility profiles of OATB, we only included the first isolate of each patient, then compared between groups for different purposes.

### 2.3 Culture and drug susceptibility testing

Isolation and drug susceptibility testing (DST) of *M. tuberculosis* was performed in the clinical laboratory of Hunan Chest Hospital, Changsha, China, a province-level reference laboratory specializing in mycobacterium detection and equipped with a full set of mycobacterium-detection facilities. Clinical specimens, including sputum, ascites, puncture fluid, pus, feces, tissues, blood, cerebrospinal fluid, and so on, were collected from TB patients and used to isolate *M. tuberculosis* by Lowenstein–Jensen (L–J) medium and (or) Mycobacterium Growth Indicator Tubes (MGIT) liquid medium, and the L–J mediums were incubated under 37°C, whilst the MGIT were incubated in the Bactec MGIT 960 mycobacterial detection instrument (Becton Dickinson Microbiology System, USA) according to manufacturer’s instructions. All *M. tuberculosis* isolates were validated by a growth test on a p-nitrobenzoic acid-containing medium (Baso, Zhuhai, China) or an MBP 64 antigen detection kit (Genesis, Hangzhou, China).

DST was performed using a Bactec MGIT SIRE kit (Becton Dickinson) in a Bactec MGIT 960 instrument following the manufacturer’s instructions. All steps were performed by well-trained technicians in a biosafety cabinet under the relevant guidelines. The reference strain H37Rv was used for quality control once a month or for each new batch of susceptibility kit. The critical concentrations in the MGIT 960 tubes were 1.0 μg/mL for streptomycin (STR), 0.1 μg/mL for isoniazid (INH), 1.0 μg/mL for rifampicin (RFP), 5.0 μg/mL for ethambutol (EMB) (17). The definitions of DR in this study were as follows: (1) Mono-DR, resistant to only one of the four drugs. (2) MDR, resistant to at least INH and RFP, the two most important first-line anti-TB drugs. (3) Poly-DR, resistant to more than one of the four drugs, but not MDR. (4) DR, resistant to at least one of the four drugs (INH, RFP, EMB, and STR).

### 2.4 Statistical analysis

Statistical analysis was done with SPSS (version 25.0, SPSS Inc., Chicago, IL, United States) and Microsoft Excel 2003 for Windows. Descriptive statistical methods were used to present the population distribution characteristics of OATB. Continuous variables were expressed as median (interquartile range, IQR), while categorical variables were described by the number of cases and proportions or rates. Chi-square test or Fisher’s exact test was used to compare different variables between groups. Binary logistic regression model was used to analyze the risk factors of DR. Results were considered statistically significant when the *p* value was less than 0.05. Statistical charts were drawn by Microsoft Excel 2003 for Windows. Map drawing was conducted by ArcGIS software (version 10.7, ESRI, Redlands, CA, USA).

## 3 Results

### 3.1 The overall epidemiology of osteoarticular tuberculosis

During the study period, a total of 288 patients with OATB were diagnosed at the Hunan Chest Hospital, after excluding 19 cases that did not conform to the case inclusion criteria, 269 patients were finally included in the study. There were 197 male cases (73.23%) and 72 female cases (26.77%), with a male-to-female ratio of 2.74:1.

**Figure 1:**
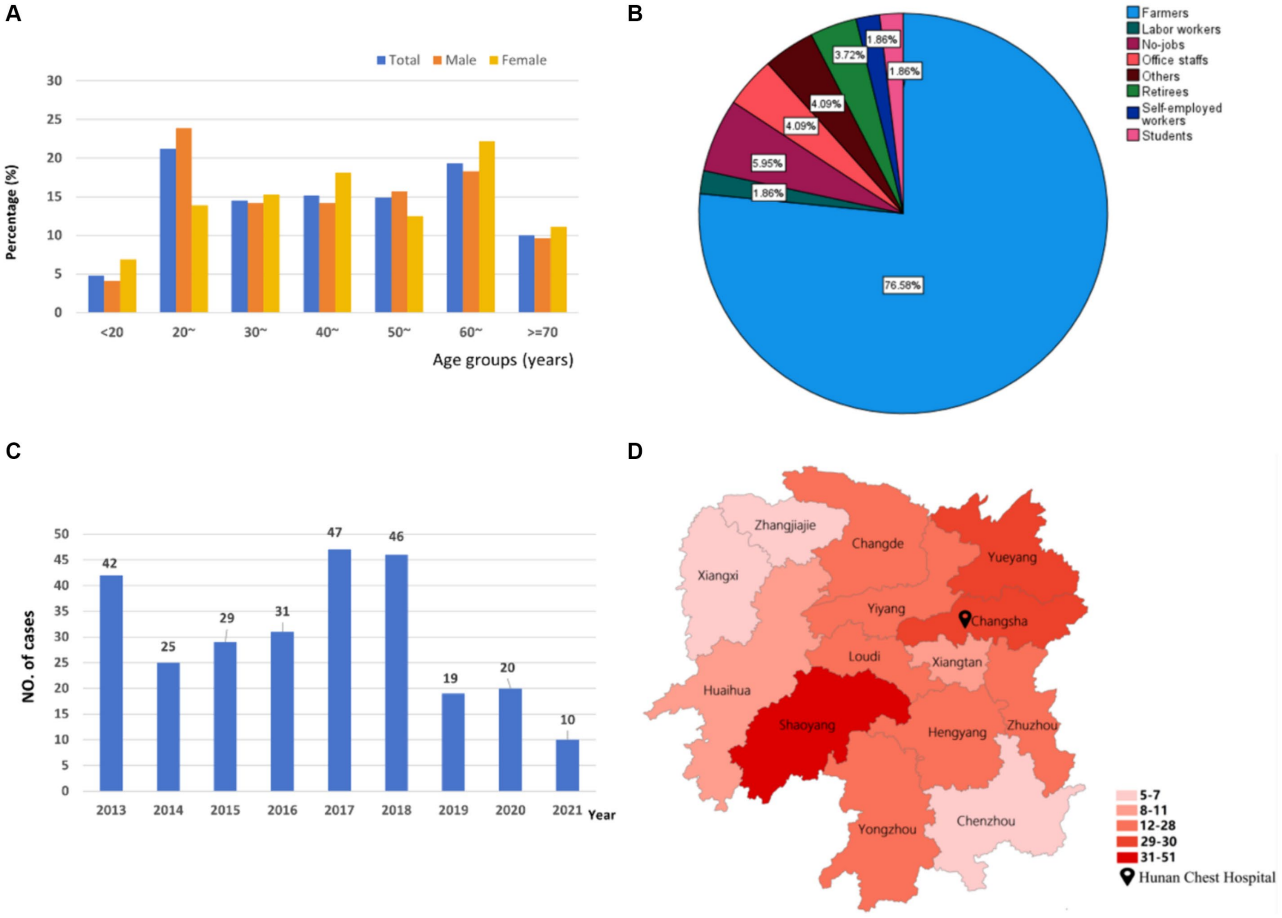
The distributions of osteoarticular tuberculosis in South-central China from 2013 to 2021. **(A)** Age distribution; **(B)** occupational distribution; **(C)** temporal distribution; **(D)** spatial distribution.

The patients aged from 5 to 82 years with a median of 45.0 (IQR: 29.0–61.5) years, most patients were in the age group 20–29 years (21.19%, 57/269), followed by 60–69 years (19.33%, 52/269), and 40–49 years (15.24%, 41/269). The age distributions of OATB in males and females were both showed typical “M” type (Figure 1A).

In terms of occupation, the cases were mainly farmers (76.58%, 206/269) (Figure 1B).

In terms of Temporal distribution, as shown in Figure 1C, the number of OATB patients was higher in 2017, 2018, and 2013 with 47 (17.47%), 46 (17.1%), and 42 (15.61%), respectively. The cases decreased by 40.5% from 2013 to 2014 and peaked during 2017–2018, followed by a large decreasing trend between 2019 and 2020.

Geographically, the majority of patients were from Shaoyang City (18.96%, 51/269), followed by Changsha City (11.15%, 30/269) and Yueyang City (10.78%, 29/269) (Figure 1D).

### 3.2 The epidemiology characteristics of the subtypes of osteoarticular tuberculosis

Among the 269 OATB patients, the exclusively OATB (without other types of TB) accounted for 54.3% (*n* = 146), OATB-EPTB (means that OATB was concurrent with other types of EPTB, not including those concurrent with PTB) accounted for 22.3% (*n* = 60), while OATB-PTB (means that OATB was concurrent with PTB whether with or without EPTB) accounted for 23.4% (*n* = 63).

#### 3.2.1 The distributions of infection sites of osteoarticular tuberculosis

Of 269 cases, the most four prevalent infection sites were lumbar vertebrae (26.02%, 70/269), multiple spinal sites (18.96%, 51/269), thoracic vertebrae (15.24%, 41/269) and lower limbs (13.38%, 36/269) (Table 1). We noticed that among the infection sites outside of the spine, lower limb (13.38%, 36/269), upper limb (4.46%, 12/269) and hip (4.09%, 11/269) were most frequent (Table 1).

**Table 1:** Distribution of affected sites of osteoarticular tuberculosis.

Infection sites	Types (%)			Total
	Exclusively OATB	OATB-EPTB	OATB-PTB	
Spine				
Cervical vertebrae	1 (0.37%)	0	0	1 (0.37%)
Thoracic vertebrae	15 (5.58%)	6 (2.23%)	20 (7.43%)	41 (15.24%)
Lumbar vertebrae	41 (15.24)	14 (5.20%)	15 (5.58%)	70 (26.02%)
Sacral vertebrae	7 (2.60%)	5 (1.86%)	2 (0.74%)	14 (5.20%)
Multiple spines^*^	28 (10.41%)	11 (4.09%)	12 (4.46%)	51 (18.96%)
Other				
Sternoclavicular	3 (1.12%)	5 (1.86%)	0	8 (2.97%)
Skull	1 (0.37%)	0	0	1 (0.37%)
Shoulder joints	4 (1.49%)	1 (0.37%)	2 (0.74%)	7 (2.60%)
Ribs	5 (1.86%)	2 (0.74%)	1 (0.37%)	8 (2.97%)
Upper limbs	10 (3.72%)	1 (0.37%)	1 (0.37%)	12 (4.46%)
Hip bones	6 (2.26%)	3 (1.12%)	2 (0.74%)	11 (4.09%)
Lower limbs	24 (8.92%)	5 (1.86%)	7 (2.60%)	36 (13.38%)
Multiple sites^#^	1 (0.37%)	7 (2.60%)	1 (0.37%)	9 (3.35%)
Total	146 (54.28%)	60 (22.30%)	63 (23.42%)	269 (100.00%)

*Means that tuberculous lesions were at least in two of four types of spine sites: cervical, thoracic, lumbar and sacral vertebrae, ^#^not include spinal tuberculosis concomitant with other osteoarticular tuberculosis. OATB, osteoarticular tuberculosis; OATB-EPTB, means that OATB was concurrent with other types of extrapulmonary tuberculosis, not including those concurrent with pulmonary tuberculosis; OATB-PTB, means that OATB was concurrent with pulmonary tuberculosis.

Of the three subtypes of OATB (Table 1), lumbar vertebrae and multiple spinal sites were the top two most common sites in both the exclusively OATB and OATB-EPTB, while thoracic vertebrae were most prevalent in OATB-PTB patients and lower limbs was the third common in the exclusively OATB.

We also compared the difference of genders, occupations and regions among the three subtypes of non-spinal OATB, but no statistical significance was found (the data was not shown).

#### 3.2.2 Characteristics of spinal tuberculosis

Of the 177 (65.80%) spinal TB cases, 51 had multiple TB lesions, whilst only 9 out of the 92 non-spinal OATB cases had multiple lesions outside of the spine, and the difference was statistically significant (*χ*^2^ = 12.65, *p* < 0.01) (Table 2).

**Table 2:** Comparison on the tuberculous lesion number between spinal and non-spinal tuberculosis.

Occurrences	Single lesion	Multiple lesions	Total	*χ* ^2^	*p*
Spinal tuberculosis	126	51	177	12.65	0.000
Non-spinal tuberculosis	83	9	92		
Total	209	60	269		

Spinal TB occurred majorly in the age groups 20–29 years old (26.55%, 47/177) and 30–39 years old (18.64%, 33/177) (Table 3). In the age groups of 20–29 and 30–39 years old, spinal TB were more common than other OATB, whilst in the age group of 60–69 years old, the result was opposite.

**Table 3:** Comparison on age between spinal tuberculosis and other types of osteoarticular tuberculosis.

Age groups (years)	Spinal TB (*n*, %)	Other OATB (*n*, %)	*χ* ^2*#*^	*p*
<20	10 (5.65)	3 (3.26)	35.31	<0.001
20~	47 (26.55)^*^	10 (10.87)^*^		
30~	33 (18.64)^*^	6 (6.52)^*^		
40~	24 (13.56)	17 (18.48)		
50~	24 (13.56)	16 (17.39)		
60~	19 (10.73)^*^	33 (35.87)^*^		
>=70	20 (11.30)	7 (7.61)		
Total	177 (100.00)	92 (100.00)		

TB, tuberculosis; OATB, osteoarticular tuberculosis. ^#^For the convenience of performing RxC Chi-square test, the age groups 40~ and 50~ years old were combined. ^*^Means that of the specific age groups, there were statistical significance on the frequency between spinal TB and other OATB by one by one comparison.

We also compared the difference of genders, occupations and regions between spinal TB and non-spinal OATB, but no statistical significance was found (the data was not shown).

### 3.3 Drug resistance profiles in patients with osteoarticular tuberculosis

#### 3.3.1 Summary of drug resistance profiles

Among the first *M. tuberculosis* isolates of 269 OATB patients, 40 (14.87%), 72 (26.77%), 17 (6.31%), and 51 (18.96%) were resistant to RFP, INH, EMB, and STR, respectively; 38 (14.13%) were MDR, 16 (5.95%) were Poly-DR, 24 (8.92%) were Mono-DR; a total of 78 (29.00%) isolates were DR.

Among the 24 mono-DR strains, mono-INH resistance was most prevalent (6.69%, 18/269). Among the 16 poly-DR strains, all isolates were resistant to both INH and STR. Among 38 MDR isolates, combining STR resistance was most prevalent (5.95%, 16/269), followed by combining EMB and STR resistance (5.58%, 15/269) (Table 4).

**Table 4:** Drug susceptibility patterns of 269 clinical *Mycobacterium tuberculosis* isolates.

Susceptibility or resistance	Exclusively OATB (%)	OATB-EPTB (%)	OATB-PTB (%)	Total (%)
Fully susceptible^*^	100 (68.49)	45 (75.00)	46 (73.02)	191 (71.00)
Resistant	46 (31.51)	15 (25.00)	17 (26.98)	78 (29.00)
Mono-resistant	15 (10.27)	3 (5.00)	6 (9.52)	24 (8.92)
H-resistant	11 (7.53)	2 (3.33)	5 (7.94)	18 (6.69)
R-resistant	1 (0.68)	0	1 (1.59)	2 (0.74)
S-resistant	3 (1.12)	1 (1.67)	0	4 (1.49)
E-resistant	0	0	0	0
Poly-resistant	9 (6.16)	3 (5.00)	4 (6.35)	16 (5.95)
MDR	22 (15.07)	9 (15.00)	7 (11.11)	38 (14.13)
HR	11 (7.53)	2 (3.33)	5 (7.94)	5 (1.86)
HRS	1 (0.68)	0	1 (1.59)	16 (5.95)
HRE	3 (1.12)	1 (1.67)	0	2 (0.74)
HRES	0	0	0	15 (5.58)

^*^Means the isolates were simultaneously susceptible to isoniazid, rifampicin, streptomycin, and ethambutol. H, isoniazid; R, rifampicin; S, streptomycin; E, ethambutol; MDR, multi-drug resistant. OATB, osteoarticular tuberculosis; OATB-EPTB, means that OATB was concurrent with other types of extrapulmonary tuberculosis, not including those concurrent with pulmonary tuberculosis; OATB-PTB, means that OATB was concurrent with pulmonary tuberculosis; Poly-resistant, these isolates were all only resistant to INH and STR.

There were 53 cases that had multiple isolates, and 48 of them kept consistent drug susceptibility patterns. Of the remaining five cases, three got additional EMB resistance, one changed from resistant to susceptible against STR, and one changed from resistant to susceptible against INH, all these changes happened during their first hospitalization which always took 2 weeks.

#### 3.3.2 Drug resistance differences among three subtypes of osteoarticular tuberculosis and between different infection sites

We compared the drug susceptibility differences among the three subtypes of OATB and between OATB in spines and that outside of spines, however, none of statistical significance on the four drugs’ resistance, MDR or DR was found (Supplementary Table 1).

#### 3.3.3 Drug resistance differences in regions, genders, age groups and occupations

According to the locations of the 14 cities in Hunan province, we divided Hunan into five areas: east (Changsha, Xiangtan, and Zhuzhou), south (Hengyang, Yongzhou, and Chenzhou), west (Zhangjiajie, Xiangxi, and Huaihua), north (Yueyang and Changde), and center (Loudi, Yiyang, and Shaoyang), then we compared the differences of six types of resistance rates among the EPTB patients from the five areas and found that the STR and RFP resistance, and MDR showed significant differences, and the west of Hunan owned the highest rates of these three resistance types (all rates were 33.33%) (Supplementary Table 1).

All comparisons on the four drugs’ resistance, MDR or DR among age groups found statistical significance (all *p* values <0.05), the 40~ years age group owned the highest rates of INH (43.90%) and RFP resistance (31.71%), and MDR (31.71%) and DR (46.34%), the 30~ years age group owned the highest rates of STR (30.77%) and EMB resistance (15.38%), while the <20 years age group also had same and highest STR resistance with the 30~ years age group (Supplementary Table 1).

None of the types of resistance rates showed differences in the distributions of gender and occupations. The details are shown in Supplementary Table 1.

#### 3.3.4 Risk factors for rifampicin/multi-drug resistant osteoarticular tuberculosis by multivariable logistic regression analysis

As RR/MDR posed a great threat to the control of TB, so we focused on its risk factors in this section. We included the two factors including age groups and regions that shown statistically significant associations with OATB in the univariate analysis into the multivariable logistic regression model. As shown in Table 5, OATB patients aged 40–49 years old (compared to those aged ≥70 years) and from the west of Hunan China (compared to those from the center of Hunan) were at risk for developing RR/MDR (odds ratios [ORs] were 5.057 and 4.942, respectively; 95% confidence intervals [CIs] were 1.009–25.342 and 1.458–16.750, respectively).

**Table 5:** Risk factors for rifampicin/multi-drug resistant osteoarticular tuberculosis by multivariable logistic regression model.

Characteristics	RR/MDR cases (*n*, %)		*p*	*OR* (95% *CI*)
	Yes	No		
Age groups (years)				
<20	2 (15.38)	11 (84.62)	0.499	2.078 (0.250–17.289)
20~	7 (12.28)	50 (87.72)	0.772	1.282 (0.239–6.880)
30~	9 (23.08)	30 (76.92)	0.156	3.290 (0.636–17.030)
40~	13 (31.71)	28 (68.29)	0.049	5.057 (1.009–25.342)
50~	6 (15.00)	34 (85.00)	0.453	1.925 (0.348–10.662)
60~	1 (1.92)	51 (98.08)	0.249	0.234 (0.020–2.761)
≥70	2 (7.41)	25 (92.59)	Reference	
Region				
East of Hunan (Changsha, Xiangtan, Zhuzhou)	7 (11.86)	52 (88.14)	0.534	1.431 (0.463–4.424)
South of Hunan (Hengyang, Yongzhou, Chenzhou)	7 (14.89)	40 (85.11)	0.312	1.785 (0.580–5.493)
West of Hunan (Zhangjiajie, Xiangxi, Huaihua)	7 (33.33)	14 (66.67)	0.010	4.942 (1.458–16.750)
North of Hunan (Yueyang, Changde)	11 (21.15)	41 (78.85)	0.066	2.627 (0.938–7.354)
Center of Hunan (Loudi, Yiyang, Shaoyang)	8 (8.89)	82 (91.11)	Reference	

OR, odds ratio; CI, confidence interval. RR/MDR, rifampicin resistance/multi-drug resistance.

## 4 Discussion

Our study first described the epidemiological and drug resistance characteristics of OATB from Hunan province, China with large samples, which provided important implications for the prevention and treatment of OATB.

The results of this study showed a much higher OATB incidence in men than in women. This finding is opposite to that in a study from Central India (18) and from South Africa (12), but consistent with the results from China (19). We also found that the occupation of OATB patients was mainly farmers (76.58%). The explanation may be that Hunan is a large agricultural province with a larger proportion of the rural population, and men bear more high labor intensity jobs than women and perform as the main workforce in society, in addition, people in rural areas usually had weaker health awareness and low nutritional level (20), and less healthcare services were available in rural area (21). It is suggested that the prevention and control of OATB in rural residents should be paid more attention.

Our results showed that OATB affected all age groups, with the peak incidence at the age groups of 20–29 and 60–69 years old. A previous study from Beijing, China reported that OATB showed a slight descent trend as age increased, the age groups of 18–39, 40–59, and ≥60 years old took proportions of 33.4, 32.7, and 26.7%, respectively, while the <18 years took only 7.1% (6), as the age group classification was not consistent with the present study, it was not easily compared between the two studies. Another study from Cape Town, South Africa showed that OATB were mostly occurred in 20–25, 1–5, and 61–65 years old (12), which partly in line with our study.

In terms of the temporal distribution, the high number of cases in 2013 could be attributed to the following two reasons. First, Hunan province implemented 10 health measures to benefit the people in 2013, including providing free anti-TB treatment for patients with active TB (22). Then, the number of patients with OATB showed a slow rising trend from 2014 to 2018, which may be related to the improvement of medical level, optimization of TB diagnostic techniques and improvement of the population’s awareness of TB. On the contrary, the explanation for the decline in the number of cases since 2019 is probably as follows: under the implementation of the “13th Five-Year Plan” for Tuberculosis Prevention and Control of Hunan province in 2018, all 14 cities (prefecture) set up hospitals for DR TB diagnosis and treatment, so the patients had more convenient shortcuts to seek medical treatment in local areas, instead of going to the provincial hospital; the number of patients decreased significantly from 2020 to 2021, which may be due to the great negative impact of the COVID-19 epidemic on the discovery, treatment and management of TB patients (23, 24).

Unlike the fluctuation in the number of cases in this study, Wang et al. found that the OATB in the southwest of China showed an upward trend from 2013 to 2021 (10). A study from central India also reported that the OATB burden was increasing from 2017 to 2021 (18). Another study on OATB between 2002 and 2019 from Oman showed that the cases were more frequent between 2015 and 2017 (25). In contrast, in Spain, the mean OATB annual incidence per million inhabitants of the first period (1997–2007) was significantly higher than that of the second (2008–2018) (6.95 vs. 5.35, *p* < 0.001) (26). These studies indicate that OATB has regional epidemiological characteristics and should be strengthened in surveillance for better control.

In terms of regional distribution, Shaoyang, Changsha, and Yueyang had higher proportions of the OATB patients. The reason may be that Shaoyang, the third largest city in Hunan Province, has a large population base. Geographically, Hunan Chest Hospital is located in Changsha, which is beneficial for patients in this area and surrounding cities like Yueyang City seeking for treatments.

Previous investigations have demonstrated that 14.37–30.0% (25, 27) of OATB patients were concurrent with PTB or (and) other types of EPTB, which was lower than our study (45.7%). The explanation may be that the samples collected in this study were from Hunan Chest Hospital, which is a designated hospital for TB treatment, the patients with PTB symptoms mostly chose to go to this hospital for treatment, leading to selection bias in the samples. Therefore, clinicians should be alert to the presence of OATB combined with PTB and assess its infectivity.

In the present study, we found that the OATB lesions distributed widely, and spinal lesion was the most prevalent (accounting for 65.8%), similar to the study from Oman (66%, 76/115) (25) and studies from China [62.03%, 165/266 (3); 64.4%, 1,988/3,086 (6)]. It is reported that spinal TB is prevalent in children and young adults (27–29), usually during the initial latent phase of the infection (28, 29). However, we found that the under 20 years old took only 5.6% (10/177), while the 20–29 years old took the highest proportion (37.5%, 47/177) of spinal TB, so our results partly confirmed the previous studies (27–29). The spinal TB is frequently asymptomatic in its early stage, but the infection usually extends into the adjacent intervertebral disc space, with contiguous infection of multiple adjacent vertebral bodies. The present study showed that 18.96% (51/269) patients had lesions in at least two of four types of spine sites: cervical vertebrae, thoracic vertebrae, lumbar vertebrae and sacral vertebrae (Table 1). Spinal TB usually and gradually contributes to bone destruction, vertebral collapse, compression of the spinal cord and nerves, and eventually symptoms such as pain, numbness, and weakness. So, the spinal TB burden must be paid more attention in its diagnosis and treatment.

Currently, DR has become a major problem in the treatment of TB. In the present study, the prevalences of MDR- and RR-OATB were 14.13 and 14.87% respectively, which were similar to that (MDR: 12.5%) from Fan et al.’ report (6), and were both twice higher than the national levels in new PTB cases in China (respectively 5.71 and 6.65%) (30). The MDR rate (14.13%) in our study was far higher than that of OATB patients from South Africa (4%, 5/125) (12) and from southwest China (7.1%, 17/241) (10). The INH resistance rate (26.77%) was the highest in the present study, which was higher than the national level among the new PTB cases (16.0%) (30); the DR rate (29.00%) was lower than the national level among the new PTB cases (34.2%) (30). A previous study from South-central China reported the resistance differences between Beijing and non-Beijing genotype *M. tuberculosis* from spinal TB with small sample sizes (56 isolates), their rates of RR (33.93%), MDR (30.36%), INH (37.5%), EMB (19.64%), and STR (26.79%) resistance in all isolates (31) were all higher than that from the present study. It was worthy to notice that, no DR difference was found among the subtypes of OATB or between spinal TB and non-spinal TB in the present study. All these clues suggested that the DR in OATB was very serious, and more precise and faster diagnosis tools and more effective treatment regimens in OATB are needed.

This study also tried to explore the risk factors of RR/MDR OATB. The results showed that OATB patients aged 40–49 years old (compared to those aged ≥70 years) and from the west of Hunan China (compared to those from the center of Hunan) were at risk for developing RR/MDR. The samples in the present study were from a provincial TB hospital in Changsha City (located in the east of Hunan), and the west of Hunan is the farthest area from Changsha City, we speculated one reason was that the OATB patients with serious, DR and not recovered from prolonged treatment were transferred to this hospital, resulting the higher resistance rates in this area. Fan et al. (6) reported that <18 years had significantly higher odds of having DR OATB compared with those aged ≥60 years (aOR, 20.778; 95% CI, 4.49–96.149), which was only partly consistent with the present study, as we only found that <20 years age group only had highest STR resistance rates, while the 40~ years age group owned the highest rates of INH (43.90%) and RFP resistance (31.71%), and MDR (31.71%) and DR (46.34%) rates, and showed as an independent risk factor of RR/MDR OATB by multivariable logistic regression.

There were some limitations in this study: first, we only included the inpatients from the Hunan Provincial Chest Hospital and the inpatients with severe symptom or DR-TB were more inclined to hospitalization, which would result in overestimated DR levels. Second, there was no available information on the categories of new cases or relapse, so that we could not report the resistance rates of each category to provide more exact clues on the management of resistant OATB.

## 5 Conclusion

In conclusion, OATB of human was more inclined to happen in males than in females, in farmers, and involve in the whole skeletal system, especially in the spine; spinal OATB always damaged multiple spinal columns. The resistance situation of OATB was serious, and people aged 40–49 years old and from west of Hunan were risk factors for developing RR/MDR-OATB. Therefore, the diagnostic technology on OATB and its susceptibility should be continuously optimized to achieve early diagnosis and effective treatment, subsequently reducing the occurrence of DR and improving the prognosis of patients.

## Data Availability

The original contributions presented in the study are included in the article/Supplementary material, further inquiries can be directed to the corresponding authors.
